# Evaluation of the palliative symptom burden score (PSBS) in a specialised palliative care unit of a university medical centre - a longitudinal study

**DOI:** 10.1186/s12904-018-0342-0

**Published:** 2018-07-07

**Authors:** Katharina Fetz, Hendrik Vogt, Thomas Ostermann, Andrea Schmitz, Christian Schulz-Quach

**Affiliations:** 1 Chair of Research Methodology and Statistics in Psychology, Department of Psychology & Psychotherapy, Faculty of Health, Witten/Herdecke University, Witten, Germany; 2 Interdisciplinary Centre for Palliative Medicine, Medical Faculty, Heinrich Heine University Dusseldorf, Düsseldorf, Germany; 3 Institute of Psychiatry, Psychology and Neuroscience; Department of Psychological Medicine, King’s College London, London, UK; 4 South London and Maudsley NHS Foundation Trust, London, UK; 5 St. Christopher’s Hospice, London, UK

**Keywords:** Palliative care, Terminal care, Symptom burden assessment, Proxy-based assessment, Physical symptoms, Psychological symptoms, Psychometric evaluation, Factor analysis, Principal component analysis, Validity, Reliability analysis, Sensitivity

## Abstract

**Background:**

The implementation of standardised, valid and reliable measurements in palliative care is subject to practical and methodological challenges. One aspect of ongoing discussion is the value of systematic proxy-based assessment of symptom burden in palliative care. In 2011, an expert-developed proxy-based instrument for the assessment of symptom burden in palliative patients, the *Palliative Symptom Burden Score* (PSBS), was implemented at the Specialised Palliative Care Unit of the University Medical Centre in Dusseldorf, Germany. The present study investigated its feasibility, acceptance and psychometric properties.

**Methods:**

The PSBS was rated by nursing staff three times a day over 5 years (*N* = 820 patients). Feasibility and nurses’ acceptance of PSBS were analysed. Structural validity was investigated by principal component analysis. Construct validity was examined via cross-validation with the *Hospice and Palliative Care Evaluation* checklist. Discriminative validity of the PSBS was analysed by means of Kruskal-Wallis test of patients’ performance score. Reliability of the PSBS was evaluated by internal consistency analysis, test-retest and split-half-reliability. Inter-rater reliability was investigated by observer agreement of nurses’ ratings of symptom burden within a day. Sensitivity to change was analysed by Wilcoxon test with repeated measures of the PSBS before and after palliative complex treatment.

**Results:**

A high degree of acceptance and the feasibility of a high-frequency proxy-based symptom burden assessment approach were demonstrated. There were low rates of missing values and no indications of the adoption of prior ratings. PSBS in its present form demonstrates good structural and construct validity (*r*_*s*_ = .27–.79, *p’s* < .001) and high sensitivity to changes in symptom burden (*p’s* < .01, except sweating), but unsatisfactory reliability (α = .41–.67; test-retest: *r*_*s* =_ .30–.88; *p’s* < .001; split-half: *r*_*s*_ = .69; *p* < .001; inter-rater: *n.s.*).

**Conclusions:**

The study presents a framework for the post hoc validation of an already existing documentation tool in palliative care. This study supports the notion that PSBS might not be reflective of an overall construct and will therefore require further development and critical comparison to other already established symptom burden instruments in palliative care.

## Background

Palliative care deals, by definition, with human beings in a very complex and difficult situation. This situation includes, for instance, multi-morbidity at a very late stage of treatment, with a multitude of different pharmacological treatments resulting in both physical and mental suffering. Consequently, patients in a palliative care setting exhibit a broad range of physical and psychological symptoms. The documentation of patients’ symptom development at short intervals by means of standardised documentation systems can improve patient care, clinical decision-making, quality assurance and evaluation of treatment delivery.

Due to the identified need for standardised documentation instruments, the last few years have seen a growing interest in the measurement of symptom development in palliative care patients. Several national and international collaborations were founded to foster and harmonise research on this topic [European PRISMA-Group [1]. A recently published consensus-paper by the European Association for Palliative Care Task Force on outcome measurement highlights the critical importance of implementing standardised, psychometrically evaluated outcome measures into the daily clinical routine in specialised palliative care units (SPCU) [2].

Nevertheless, there are several practical and methodological challenges to consider when examining methods of documenting symptoms [3]. The first challenge is determining an adequate outcome measure for the end-of-life setting and the frequency of measurement. The numerous existing outcome measurement systems were not developed for palliative care populations, and most of them have not yet been validated [4]. Given the high temporal variability in patients’ symptom presentation, there is no consensus on the appropriate frequency of symptom measurement [4, 5]. Even though the self-report is considered the gold standard to obtain information on patient symptom burden [4], many palliative care patients are not able to complete questionnaires or answer questions due to fatigue, decreased alertness or delirium [6]. For other patients, a high-frequency self-assessment approach might constitute an undue burden. Additionally, being confronted with a terminal disease and the prospect of proceeding towards their personal death results in severe distress and a broad range of affective reactions, which in turn cause various coping and self defence mechanisms [7, 8]. Those mechanisms might also lead to bias with regard to not reporting symptoms. Consequently, especially in the end-of-life setting, proxy-based symptom documentation appears to be a promising additional source of information to complement self-reported measurement instruments. Because high-frequency proxy-based symptom measurement approaches always entail an increased workload for hospital staff, the successful implementation of such an approach depends to a great degree on its practical feasibility in daily clinical routine and its acceptance by nurses and physicians [9].

Currently, only a few validated proxy-based assessment tools for symptom burden in palliative care patients are available in the German language: The *Basic Documentation for Psycho-Oncology* [10], the *Hospice and Palliative Care Evaluation* checklist [HOPE, [11] and the *Edmonton Symptom Assessment System* [ESAS, [12].

The *Basic Documentation for Psycho*-*Oncology* focuses on the psycho-social burden of cancer patients. HOPE measures the symptom burden of the previous three days of palliative patients. ESAS was originally developed as a self-assessment tool for symptom burden in cancer patients but is also used as a proxy-based assessment tool in some cases.

Nevertheless, a large number of SPCUs and hospices still use non-validated, unpublished, self-administered and non-expert-developed documentation tools to assess patients’ symptom burden. To acquire additional knowledge regarding outcome measures in palliative care, it would be advisable to evaluate these instruments in terms of their psychometric validity and to share experiences in the implementation of such approaches with the scientific community in addition to palliative care practitioners.

One outcome instrument used within the interdisciplinary palliative care centre at the university hospital Duesseldorf (see methods for details) is the *Palliative Symptom Burden Score* [PSBS, [13], which measures physical and psychosomatic symptom burden. The PSBS items are *alertness*, *confusion*, *restlessness/anxiety*, *sweating*, *weakness*, *nausea*, *vomiting*, *dyspnoea*, *coughing*, *pain* and *constipation*. Symptom intensity is rated on a ten-point verbal rating scale three times a day by nursing staff. The data collected by PSBS were first reported in a study on high-dependency palliative care patients dying in a tertiary hospital inpatient unit [13]. To date, no studies have examined the psychometric properties of PSBS. The tool was heuristically developed by clinical experts in palliative care. The most frequent symptoms in palliative care patients according to experts’ clinical impressions were included as items for the instrument (see Table 1); there has been no further psychometric validation.

**Table 1:** Setting of the SPCU

Physicians in clinical service	5.50^a^
Deputies	2.00^a^
Medical specialists	4.00^a^
Ward physicians	3.00^a^
Nursing staff	18.00^a^
Physiotherapists	2.00^a^
Art therapists	2.00^a^
Clergy	2.00^a^
Psychologists	1.00^a^
Social service	1.00^a^
Beds	8.00^b^
Utilisation of bed capacity ^d^	84.70^c^
Ward round	Daily
Deputy ward-round	Twice a week
Team meeting	Daily
Interdisciplinary case conference	Weekly
Case supervision	Bi-weekly
Team supervision	Monthly
Physicians in attendance	7:30–19:30
Physicians’ on-call duty	19:30–7:30

^a^Number of jobs

^b^Number of beds

^c^%

^d^84.7% of beds are occupied on average at any given time

In 2011, the SPCU of the University Medical Centre in Dusseldorf, Germany implemented a proxy-based measurement instrument embedded in the electronic patient record (EPR) for high-frequency assessments of symptom burden in palliative patients [14]. Physical and psychological symptom burden is now assessed by means of the PSBS [8]. To our knowledge, no studies have yet been conducted implementing a longitudinal proxy-rated, high-frequency assessment system into daily clinical care. Considering the importance of such an approach for the quality assurance of treatment delivery and clinical evaluation of therapy outcomes, the present paper intends to share empirical knowledge concerning the feasibility and acceptance of longitudinal high-frequency proxy-based assessments of symptom burden in palliative patients. Given the demand for reliable and valid instruments [2], this study reports data concerning the psychometric properties of the PSBS and presents a framework for the validation of expert-created tools in palliative care. Based on the experience gained during the implementation process, the paper also presents practical and useful recommendations for the development, implementation and evaluation of proxy-based assessments in SPCUs.

## Methods

### Study design

This study was an observational cohort study with a retrospective analysis of longitudinal data on symptom burden assessment in an inpatient palliative care setting. The study reporting follows the STROBE [15] guidelines for reporting observational cohort studies. This study was approved by the Ethics Committee of the Medical Faculty of Heinrich Heine University Dusseldorf, Germany (protocol number 5287, approved 09 November 2015).

### Setting

The Interdisciplinary Centre for Palliative Medicine is a SPCU at a university hospital in an urban area of Germany. It offers inpatient palliative care treatment at a ward with 8 beds. Furthermore, there is a liaison service for inpatients at other hospitals. A detailed overview of the setting, i.e., the SPCU Dusseldorf, is presented in Table 1. The data for this study were only collected from inpatients of the SPCU (not from inpatients of other hospitals).

### Implementation

High-frequency proxy-based symptom assessment by means of the PSBS was implemented in August 2011. To train nurses in the utilisation of the new documentation system and to foster their acceptance for the new approach, a training course was offered. Subsequently, a pilot phase was conducted in which the nurses were asked to evaluate the documentation system and share their experiences with it in daily clinical routine. As a result of the nurses’ feedback, the user interface was adjusted to enhance its ease-of-use.

### Data

#### Dependent variables

##### The palliative symptom burden score

The PSBS was developed as a high-frequency documentation tool for medical professionals to measure the symptom burden of palliative care patients. Symptom burden is rated three times daily, measuring the last 8 h. An overview of its 10 items and their assessment is given in Table 2. Symptom burden indicators were originally developed by an expert panel of two palliative care physicians and one senior palliative care nurse in a heuristic process including a narrative literature search and iterative discussion. The original development of the instrument took place in one specialised palliative care centre in Berlin during the pioneer phase of palliative medicine in the 1990s in Germany and did not follow traditional tool development guidelines. The final set of items used for the instrument was based on expert opinion and had not initially been tested in a pilot phase. Patients or carers were not involved in the development phase. The items in the PSBS represent the most common symptoms of patients in SPCUs as defined by the original expert panel: *alertness*, *confusion*, *restlessness/anxiety*, *sweating*, *weakness*, *nausea*, *vomiting*, *dyspnoea*, *coughing*, *pain* and *constipation*. Each symptom is measured with one item. The intensity of the symptom is rated by means of a five-point verbal rating scale ranging from zero points (no symptom burden) to five points (strong symptom burden). Pain was rated via a 10-point verbal rating scale ranging from zero points to ten points. For reasons of comparability to the other items and for the statistical analyses, it was converted into a 5-point verbal rating scale after data collection. *Constipation* was originally measured dichotomously, reported as *yes* or *no*. Consequently, this item was excluded from the PSBS because conversion into a 5-point verbal rating scale was not possible. In total, the PSBS consists of 11 items. Symptom assessment and operationalisation of the items are described in Table 2. Overall symptom burden is reflected by the sum of single items (Min = 0; Max = 44).

**Table 2:** Items and operationalisation of the PSBS

Item	Operationalisation				
Score	0 No impairment	1 Light impairment	2 Moderate impairment	3 High impairment	4 Severe impairment
1. Alertness	No impairment	Fatigue during the day	Occasional sleep cycles during the day	Mainly sleep cycles during the day	Somnolence
2. Confusion	No impairment	Patient feels too weak for temporal and local orientation	Temporary disorientation	Patient feels high impairment, depressive reaction	Severe confusion, helplessness
3. Restlessness/anxiety	No impairment	Occasional, patient can express the cause	Frequently, care needed	Despite medication, paroxysmal	Pronounced restlessness, panic, suicidal tendencies
4. Sweating	No impairment	Occasional, after activity	Attacks of sweating accompanied by dizziness	Pronounced sweating, laundry change needed	Persisting, quickly recurring sweating
5. Weakness	No impairment	Daily routine possible with great effort	Daily routine possible with great effort and periods of rest	Help needed for activities of daily living	Care-dependent
6. Nausea	No impairment	Temporary with loss of appetite	Occasional, but multiple times per day	High impairment, retching; emesis	Persisting nausea, for hours
7. Vomiting	No impairment	Occasional vomiting after medication or ingestion	Spontaneous vomiting	Multiple vomiting (per stint)	Excruciating vomiting with persistent nausea
8. Dyspnoea	No impairment	Dyspnoea on exertion	Dyspnoea on slight exertion	Dyspnoea at rest	Paroxysmal dyspnoea anxiety, medication needed
9. Coughing	No impairment	Productive cough	Dry cough	Persisting cough	Excruciating cough, pain, fear of suffocation
10. Itching	No impairment	Occasional	Slight itching	Excruciating itching; medication needed	Excruciating itch despite medication
11. Pain	Severity 1–2	Severity 3–4	Severity 5–6	Severity 7–8	Severity 9–10
12. Constipation^a^	No	Yes	--	--	--

^a^Constipation was excluded from the analyses

Regarding component structure, it was proposed by the authors that the items *alertness*, *confusion*, *restlessness/anxiety* and *weakness* may constitute a component indicating a psychosomatic symptom complex subscale. In addition, *nausea* and *vomiting* were allocated to a gastrointestinal subscale and *dyspnoea* and *cough* to a respiratory subscale. There were no further groupings regarding *pain, sweating* and *itching*. The authors therefore expected six components of symptom burden.

##### Hope

The symptom and problem checklist HOPE [11, 16] consists of 16 items for the documentation of symptom burden of the previous 3 days. Eight items (*pain*, *nausea*, *vomiting*, *dyspnoea*, *constipation*, *weakness*, *loss of appetite*, *tiredness*) measure physical symptoms, four items (*feeling depressed*, *anxiety*, *tension*, *disorientation/confusion*) measure psychological issues, two items (*wound care*, *activities of daily living*) measure nursing issues, and two items (*organisation of care*, *overburdening of the family*) measure social issues [16]. Additionally, one free entry is provided for possible further issues, e.g., symptoms that are not assessed in the instrument. The symptom intensity is measured on a 4-point verbal rating scale (0 = no, 1 = mild, 2 = moderate, 3 = severe). Sum scores are calculated for the four subscales and a global sum score ranging from a minimum of zero points to a maximum of 51 points.

HOPE’s item structure is similar to the PSBS: *anxiety, confusion, weakness, nausea, vomiting, dyspnoea* and *pain* are measured in both HOPE and PSBS. HOPE’s item *tension* can be compared to PSBS’ item *restlessness.* In both instruments, symptom burden is rated via verbal rating scales (HOPE: 4-point Likert-Scale; PSBS: 5-point Likert-Scale). Due to these similarities, HOPE was chosen for cross-validation and investigation of the construct validity of PSBS. Nevertheless, the instruments are not completely interchangeable. While PSBS covers *alertness* as an important cognitive parameter within psychological symptoms, HOPE includes feeling *depressed* as an important psycho-affective component, which is not measured by PSBS. Additionally, HOPE includes items for loss of *appetite* and *tiredness*, (symptoms), which are not included in PSBS, while PSBS includes *coughing*, *itching* and *sweating*. Thus, PSBS and HOPE can be considered two distinct measures for symptom burden measurement that have main overlaps but also cover different aspects of symptom burden in palliative care patients.

##### The ECOG scale of performance status

The Eastern Cooperation Oncology Group (ECOG) scale of performance status [17] is a widely used prognostic tool to quantify functional status in cancer patients. In palliative care, it has also been used to report the functional status of non-cancer patients with life-limiting illness [18]. The ECOG describes patients’ functional status regarding ambulatory status and need for care. The scale categorises functional status via five symptom burden classes (0–4). A score of zero indicates normal activity; a score of one point indicates that the patient is able to walk and that light activity is possible. A score of two points means the patient is < 50% bedridden, with self-care being possible; a score of three points means the patient is > 50% bedridden with limited self-care capability, while a score of 4 points indicates the patient is completely bedridden and in need of care [19].

#### Independent variables

##### Palliative complex treatment

Between day one and day seven of treatment at the SPCU, patients received a specialised palliative complex treatment that included a set of interventions performed by palliative care professionals focusing on patient stabilisation and the reduction of symptom burden.

### Data collection

Data collection was performed between August 2011 and August 2015. Symptom burden assessment by means of the PSBS was conducted three times a day by trained palliative care nurses of the SPCU. The results were documented digitally via a standardised documentation interface. An assessment took two to 3 min. HOPE and ECOG were measured on admission and at discharge. For deceased patients, assessments for PSBS, HOPE and ECOG were performed post-mortem by nurses within a day after death. Among the patients, 476 (58%) died at the SPCU, 298 (36.30%) were discharged, 27 (3.30%) were moved to another ward within the university hospital and 9 (1.10%) were moved to another institution (e.g., hospice). Patients’ palliative stage was reported for day one of admission for those patients in whom initial assessment of performance stage and clinical survival prediction was deemed reliable. A majority of patients needed a longer period of assessment and were discussed during our weekly multidisciplinary team meetings to improve prognostic accuracy, as suggested by White et al. [20].

### Sample

Sample characteristics regarding age, sex, diagnosis group, palliative stage and ECOG performance status are shown in Table 3.

**Table 3:** Sample characteristics (N = 820)

Characteristic	Index
Age^a^	67.00 (17.00-114.00; 14.00)
Sex^b^	
Female	420 (51.20)
Male	400 (48.80)
PCU-stay^a^	
Days	10.54 (0.00 – 53.00; 9.12)
Hours	266.15 (0.00 – 1275.00; 219.37)
Diagnosis group^b^	
Cancer	690 (84.00)
Non-cancer	59 (7.20)
Previous cancer	68 (8.30)
Missing	3 (0.50)
Palliative stage^cb^	
Rehabilitation stage	34 (4.15)
Early terminal stage	129 (15.73)
Late terminal stage	152 (18.54)
Final stage	28 (3.41)
Missing	477 (58.17)
ECOG Performance Status Scale^cb^	
0 Normal activity	4 (0.50)
1 Able to walk, light activity possible	45 (5.50)
2 Self-care possible; able to walk < 50% of daytime	20 (2.40)
3 Limited self-care; < 50% bedridden	220 (26.80)
4 In need of care; bedridden	399 (48.70)
Missing	132 (16.10)

^a^Mean, (range; *SD*)

^b^*n* (%)

^c^At admission

### Statistical analyses

Patient data were extracted from the clinic’s electronic medical records and anonymised before transferring the data into SPSS. All statistical analyses were performed using IBM SPSS 22 for Windows (IBM Corp. in Armonk, NY). The data were checked for plausibility prior to inferential analyses. Descriptive statistics are reported.

For each analysis, the data timepoint is reported, whereas the first number indicates the day of data collection and the second number indicates the daytime (morning, noon, evening). For example, t1_3 is day 1 (admission), measure 3 (evening) and t7_1 is day 7 (1 week after admission), measure 1 (morning). We used measure 3 (evening) for the analyses wherever possible due to low rates of missing values. For comparisons within a day, data for day 7 instead of day 1 were used for the same reason.

### Feasibility and acceptance of the PSBS

To evaluate the feasibility and acceptance of the PSBS, high-frequency documentation data were investigated regarding rates of missing values. In addition, the data were checked for potential bias caused by the adoption of prior ratings by means of the Kendall-*W* coefficient of concordance [21]. High and significant Kendall-*W* values and significant results were assumed to be an indicator of systematic adoption of prior ratings.

### Validity

#### Structural validity

PSBS’ structural validity data from timepoint t1_3 was analysed because of a low rate of missing values. To investigate the structural validity of the PSBS, a principal component analysis (PCA) with a cut-off criterion of 6 principal components was conducted. Although PCA is not a factor analysis, it is the most frequently used approach for data reduction in psychology [22]. Analyses were performed in accordance with the procedure suggested by Klopp [22]:

##### Suitability of the data for PCA

Prior to analysis, the data were controlled for adequacy to perform a principal component analysis using the Kaiser-Meyer-Olkin measure of sampling adequacy (KMO) and Bartlett’s test of sphericity. KMO-values > .05 [23] and significant Bartlett’s test results were taken as indicators of the adequacy of the data for PCA.

##### Number of components

The main goal of principal component analysis is to determine a component structure that is stable concerning the performed method of component extraction and rotation and replicable in other conditions. In subjective assessment methods, such as scree plot analysis [24], there are some objective procedures. A criterion for estimation of the number of components to be extracted is the replicability of the component structure. Therefore, the dataset was split into two random samples, and two principal component analyses were performed on each random sample with a cut-off criterion of the proposed number of components. The resulting two component loading matrices were then compared with each other by calculating Tucker’s coefficient of congruency [25, 26] as follows:

$$ {C}_{jk}=\frac{\sum_{i=1}^p{a}_{ij}\cdot {b}_{ik}}{\sqrt{\left({\sum}_{i=1}^p{a}_{ij}^2\right)\cdot \left({\sum}_{i=1}^p{b}_{ik}^2\right)}} $$

where a_ij_ represents the loading of variables i on component j of the first component loading matrix, and b_ik_ is the loading of variables i on component k of the second component loading matrix. The resulting coefficient C may have values between − 1 and + 1, which can be interpreted similarly to Pearson’s correlation coefficient [27]. Values of Tucker’s congruency coefficients > .80 are assumed to be indicators of good replicability of the component structure [26].

##### Interpretability and rotation of the principal components

To facilitate the interpretability of the component solution, we chose the orthogonal rotation method varimax. The aim of the varimax rotation method is to achieve a simple structure of the component solution, which means that some variables load very high on one component, while other variables load very low. Thus, the variance of the squared component loadings is maximised [28].

##### Significance of component loadings

After facilitation of interpretability by means of varimax rotation, the variables that are used for the interpretation of a component must be determined. In accordance with the rule proposed by Gorsuch [29], only variables with component loadings < .30 were assumed to correspond to a component. We further considered the general rule of Guadagnoli and Velicer [30]: if fewer than 10 variables have a component loading > .40, then the sample size must be greater than 300 persons.

### Construct validity

Due to its comparable item structure and similar outcome measure, the construct validity of the PSBS was investigated via cross-validation with the HOPE checklist. HOPE subscales *nursing problems* and *social problems* were excluded because there were no similar subscales in the PSBS. Spearman’s rank correlation was calculated for sum scores and subscales of the PSBS and HOPE. Because HOPE does not include a gastrointestinal and respiratory symptom complex component, no analysis concerning these PSBS subscales was performed. Consequently, further analyses were performed on single item levels, Significant positive correlations were assumed to be indicators of good construct validity.

### Discriminative validity

The discriminative validity of the PSBS was investigated using two nonparametric analyses of variance using the Kruskal-Wallis test [31] with ECOG performance status stages as independent and the PSBS sum score at t1_3 and t7_3 as dependent variables.

### Reliability

The internal consistency of the PSBS subscales was tested by Cronbach’s alpha.

In accordance with [32], values > .70 were taken as indicators of acceptable internal consistency. Additionally, the split-half reliability for the whole test was calculated using the odd-even method. Spearman-Brown coefficients [33] are reported. The test-retest reliability was evaluated by Spearman’s rank correlation of PSBS sum scores and subscales within a day (t7_1 morning and t7_3 evening) and within a week (at t1_3 and after 1 week of treatment t7_3). To assess PSBS inter-rater reliability, intermediate measurements of different nurses during a day (t7_1, t7_2, t7_3) were examined using Kendall’s *W* concordance coefficient [21].

### Sensitivity to change

To investigate the sensitivity of the PSBS to changes in patients’ symptom burden as a consequence of treatment interventions, the sum scores of the PSBS were evaluated with respect to significant mean differences pre- (t1_3) and after complex palliative treatment (t7_3) using the Wilcoxon test with repeated measures [34]. The level of significance was Bonferroni-adjusted to *p* < .01. Only patients who completed the palliative complex treatment were included in the analysis (*n* = 514). Patients who died within the first week and did not complete treatment were excluded from the analysis.

## Results

### Feasibility and acceptance of the PSBS

Analyses showed a high degree of acceptance of the PSBS implementation by the specialised palliative care nurses. The rates of missing values in the PSBS documentation were low (0.32%).

### Descriptive PSBS and HOPE

The overall mean PSBS score was 12.21 (*SD* = 4.40; *n* = 784, missing = 36). Female patients had a mean PSBS score of 12.50 (*SD* = 4.45; *n* = 381. Male patients’ mean PSBS score was 11.94 (*SD* = 4.33, *n* = 403). Cancer patients had a mean PSBS score of 12.88 (*SD* = 4.33, *n* = 664), previous cancer patients 12.78 (*SD* = 4.29, *n* = 64) and non-cancer patients 13.78 (*SD* = 4.97, *n* = 56). Patients’ overall mean HOPE score was 23.80 (*SD* = 5.48, *n* = 782, missing = 38). Female patients had a mean HOPE score of 23.83 (*SD* = 5.99, *n* = 378) and male patients of 23.33 (*SD* = 5.98, *n* = 404). Cancer patients had a mean HOPE score of 23.66 (*SD* = 6.08, *n* = 658), previous cancer patients of 23.68 (*SD* = 5.06, *n* = 67) and non-cancer patients of 25.55 (*SD* = 5.62, *n* = 57).

### Validity

#### Structural validity

Data were adequate for PCA with a Kaiser-Meyer-Olkin criterion of .61 and a significant Bartlett’s test of sphericity (χ^2^(55) = 1020.80 *p* < .0001). PCA revealed six main component solutions explaining 73.12% of the variance. Analysis of component replicability revealed values of Tucker coefficients of congruence greater than .80 for all of PSBS’ scales, indicating good replicability of the main components. Principal components, explained variance, Tucker coefficients of congruence, corresponding items and component loadings are presented in Table 4.

**Table 4:** Component solution of the PSBS

Main component^a^	Expl. variance (%)^bc^	C ^d^	Items	Factor loading
1. Psychosomatic symptom complex	18.34	.97	*Alertness*	.74
			*Confusion*	.79
			*Restlessness/ anxiety*	.51
			*Weakness*	.66
2. Gastrointestinal symptom complex	14.15	.97	*Nausea*	.87
			*Vomiting*	.88
3. Respiratory symptom complex	11.89	.98	*Dyspnoea*	.76
			*Coughing*	.82
4. Sweating	10.17	.82	*Sweating*	.91
5. Pain	9.40	.87	*Pain*	.84
6. Itching	9.16	.85	*Itching*	.93

^a^Principal component analysis; extraction criterion = 6 main components

^b^Rotation method: varimax rotation, Kaiser-normalisation

^c^Cumulative explained variance = 73.12%

^d^Component replicability: Tucker’s coefficient of congruency

#### Construct validity

There was a significant positive correlation of PSBS on admission with HOPE scores on admission (*r*_*s*_ = .58; *p* < .001) and at discharge (*r*_*s*_ = .54; *p* < .001). The *psychological problems* scale of HOPE correlated significantly with the psychosomatic symptom complex of the PSBS on admission (*r*_*s*_ = .43; *p* < .001) and at discharge (*r*_*s*_ = .28; *p* < .001). As principal component analysis did not reveal a physical symptom burden complex component for the PSBS, no correlations concerning the physical problems subscale of HOPE were calculated. Single item correlations of PSBS and HOPE checklist revealed positive significant correlations ranging between *r*_*s*_ = .48 and *r*_*s*_ = .79 on admission and between *r*_*s*_ = .18 and *r*_*s*_ = .61 (all *p*-values < .001) 1 week after admission. The single item correlations of the PSBS and the HOPE checklist are presented in Table 5.

**Table 5:** Convergent validity

Item PSBS	Item HOPE	*r*_*s*_ admission	*n*	*r*_*s*_ discharge	*n*
Restlessness/ anxiety	Anxiety	.48	763	.23	757
Restlessness/ anxiety	Tension	.43	763	.18	756
Confusion	Confusion	.79	762	.53	756
Weakness	Weakness	.46	766	.61	758
Nausea	Nausea	.69	767	.27	758
Vomiting	Vomiting	.61	766	.38	758
Dyspnoea	Dyspnoea	.61	766	.53	758
Pain	Pain	.77	762	.34	754

All *p*-values < .001

Two nonparametric analyses of variance using the Kruskal-Wallis test revealed significant differences in PSBS sum scores for patients in different subgroups of ECOG on admission (χ ^2^ (4) = 121.91; *p* < .001) and 1 week after admission (χ ^2^ (4) = 57.68; *p* < .001). The mean sum scores for each ECOG group and measurement point are presented in Table 6.

**Table 6:** Discriminative validity

ECOG Performance Status	PSBS sum score t1^ab^	PSBS sum score t2^ac^
0. Normal activity	7.75 (3.86)	8.75 (6.18)
1. Able to walk, light activity possible	10.06 (4.13)	8.80 (3.38)
2. Self-care possible; able to walk < 50% of daytime	7.36 (3.05)	11.65 (3.41)
3. Limited self-care; < 50% bedridden	10.35 (3.33)	11.70 (4.36)
4. In need of care; bedridden	12.61 (3.68)	12.90 (3.70)

^a^Mean (*SD*)

^b^t1_3 (= measure 3, day 1): (χ 2 (4) = 121.91; *p* < .001)

^c^t7_3 (day 7, measure 3 (evening): (χ 2 (4) = 57.68; *p* < .001)

### Reliability

The Cronbach’s alpha coefficients for the PSBS sum score and the PSBS subscales did not meet the criterion of acceptable internal consistency (> .70). The coefficients are presented in Table 8. The split-half reliability was investigated using the odd-even method. The results were adjusted using the Spearman-Brown-formula, revealing a coefficient of .69. Spearman’s rank correlation of PSBS sum scores on admission and PSBS sum scores 1 week after admission revealed a significant positive moderate correlation (*r*_*s*_ = .55; *p* < .001). Correlations and *p*-values for the PSBS subscales are shown in Table 7. Analyses of inter-rater reliability revealed poor and non-significant values for all items but *confusion* (Kendall’s *W* = .01; χ^2^ (2) = 9.97; *p* < .01). *Pain* marginally missed the level of significance (Kendall’s *W* = .01; χ^2^ (2) = 5.92; *p* = .05). The results of inter-rater-reliability analyses therefore indicated no hints for systematic adoption of prior ratings. Kendall’s *W*, chi-square- and *p*-values for each item are shown in Table 8.

**Table 7:** Reliability coefficients

Scale	Items	Internal consistency^b^	Test-retest reliability^d^ Interval = one day^f^	*n*	Test-retest reliability^d^ interval = one week^g^	*n*
1. Psychosomatic symptom complex	4	.67	.88	741	.66	513
2. Gastrointestinal symptom complex	2	.67	.72	741	.37	514
3. Respiratory symptom complex	2	.41	.87	741	.67	513
4. Sweating^a^	1	-	.79	739	.30	506
5. Pain^a^	1	-	.55	736	.30	512
6. Itching^a^	1	-	.81	741	.53	513
Sum score^e^	11	.53	.83	741	.55	784

^a^Single item scales, no analyses of internal consistency performed

^b^Cronbach's alpha

^d^*r*_*s*_ Spearman’s rank correlation; all *p*-values <.001

^e^Split-half-reliability: *r*_*s*_ = .69; *p* < .001

^f^Interval of one-day time points: t7_1 (day 7, measure 1 (morning)) and t7_3 (day 7, measure 3 (evening))

^g^Interval of one-week time points: t1_3 (day 1, measure 3 (evening)) and t7_3 (day 7, measure 3 (evening))

**Table 8:** Inter-rater reliability

PSBS	Kendall’s *W*	χ^2^ (*df*)	*n*	*p*
Single items				
1. Alertness	0.003	3.55 (2)	513	.17
2. Confusion	0.010	9.97 (2)	513	.01
3. Restlessness/ anxiety	0.000	0.13 (2)	513	.94
4. Sweating	0.003	3.15 (2)	506	.21
5. Weakness	0.000	0.01 (2)	513	.99
6. Nausea	0.002	1.67 (2)	514	.43
7. Vomiting	0.001	1.22 (2)	514	.54
8. Dyspnoea	0.003	3.04 (2)	513	.22
9. Coughing	0.002	1.65 (2)	513	.44
10. Itching	0.000	0.40 (2)	513	.82
11. Pain	0.006	5.92 (2)	512	.05
Sum score	0.000	0.40 (2)	514	.82

### Sensitivity to change

The Wilcoxon test with repeated measures showed significant differences before and after palliative complex treatment for all PSBS subscales and sum scores except *sweating* (z = − 0.34; *p* = .73). The mean PSBS subscales and sum scores before and after palliative complex treatment with corresponding *z*- and *p*-values are presented in Table 9.

**Table 9:** Sensitivity to change

Scale	PSBS t1_3^b^	PSBS t7_3^b^	*n*	*z*	*p*
1. Psychosomatic symptom complex	6.81 (3.16)	6.94 (3.00)	513	-6.35	< .01
2. Gastrointestinal symptom complex	0.82 (1.30)	0.51 (0.96)	514	-5.47	< .01
3. Respiratory symptom complex	1.84 (1.58)	1.79 (1.45)	513	-2.41	< .01
4. Sweating^a^	0.35 (0.76)	0.24 (0.60)	506	-0.34	.73
5. Pain^a^	1.99 (0.95)	1.70 (0.80)	512	-2.85	< .01
6. Itching^a^	0.21 (0.55)	0.27 (0.61)	513	-3.14	< .01
Sum score	12.21 (4.40)	11.70 (4.13)	514	-2.02	< .01

^a^Single item scales

^b^Mean (*SD*)

## Discussion

The aim of the present study was to report the implementation, acceptability and feasibility of a high-frequency proxy-based symptom assessment instrument in palliative care, to describe data concerning the psychometric properties of the instrument and to present a framework for the evaluation of such an approach. Systematic proxy-based assessment of symptom burden in palliative care obtained by nurses can be similar in accuracy to patient-reported outcomes and has special value in low-functioning or confused patients [9, 35].

### Feasibility and acceptance

Since its implementation in 2011, the PSBS has been integrated into daily clinical routine at the SPCU at the University Medical Centre in Dusseldorf, Germany. Symptom burden was successfully documented three times a day, and further analysis showed a low rate of missing values and no hints of adoption of prior ratings. We would argue that this finding can be interpreted as two indicators of the acceptability of this instrument, but interviews with nurses who conduct their daily assessments with PSBS are needed to confirm this preliminary finding. In summary, successful implementation of PSBS and the quantitative analysis of nurses’ ratings provide some evidence for the feasibility of a high-frequency proxy-based symptom documentation approach in a SPCU. To gain a more detailed understanding of PSBS’ feasibility and acceptance in clinical practice, an implication for further research is to conduct qualitative assessments, e.g., by means of qualitative interviews with nursing staff.

### Psychometric properties

#### Validity

Because PSBS as an expert-developed documentation instrument has not yet been validated, another aim was to report data concerning the psychometric properties of this instrument. PCA revealed six main component solutions, including three multiple-item subscales (psychosomatic symptom complex; gastrointestinal symptom complex and respiratory complex) and three single-item scales (*pain*, *itching*, *sweating*). Considering the large amount of explained variance, the psychosomatic symptom complex appears to be a very relevant aspect of palliative patient symptom burden. This result is in agreement with former studies highlighting the importance of psychological symptoms in palliative care patients [36].

Several different methods are used for data reduction. Common factor analysis (CFA) and principal component analysis (PCA) are widely used multivariate techniques for this purpose [37]. According to Widaman [38], “the final word on comparisons between CFA and PCA has not yet been written” (p. 201). In the present study, we chose PCA for data reduction and evaluation of PSBS’ structural validity because nonzero PCA loadings are higher and more stable than nonzero common factor analysis loadings and are closer approximations of the true factor loadings than the loadings produced by common factor analysis [37]. An implication for further research is to further evaluate PSBS’ latent factor structure by structural equation modelling.

PSBS and HOPE sum scores showed a positive moderate significant correlation on admission and at discharge, indicating good construct validity of the PSBS. The aspect of moderate correlations implies that both instruments measure similar objectives but are not redundant, potentially due to the slightly different items. The psychosomatic subscales of the PSBS and HOPE show moderate positive significant correlations on admission and at discharge, which may be because both instruments cover different aspects of mental symptom burden. While the PSBS measures *alertness* and *weakness* as important mental symptoms of palliative care patients, HOPE covers *depression*, which is of no less importance. The results of single item correlations of the PSBS and HOPE support the construct validity of the PSBS. Interestingly, the strength of the correlations decreases at the second point of measurement (discharge), which may be caused by HOPE post-mortem ratings for deceased patients. Univariate analysis of variance showed significant ECOG subgroup differences in mean PSBS sum scores, demonstrating a good discriminative validity of the PSBS regarding different intensities of symptom burden.

#### Reliability

Analyses of the internal consistency of PSBS subscales revealed below cut-off results for all subscales. Whereas acceptable reliability was almost met by the psychosomatic and gastrointestinal symptom complexes, values for the respiratory symptom complex showed poor internal consistency. These indicators do not support the use of the proposed subscales for symptom assessment in the current instrument. It might be best to measure symptom burden on a single item level. The sum score for PSBS should not be used because it does not appear to be reliable.

The split-half reliability of the PSBS also slightly missed the criterion of acceptable reliability. Analysis of test-retest reliability showed a moderate correlation between PSBS sum scores on admission and 1 week later. A correlation of .70 is an indicator of fair test-retest reliability, but this value highly depends on the interval between the points of measurement. In terms of a state-like symptom burden that is subject to frequent fluctuations within a single day, an interval of 1 week may have been too short to detect good test-retest reliability.

The results of test-retest reliability of PSBS subscores indicated a difference in stability between symptom burden subscores. The psychosomatic symptom complex and the respiratory symptom complex appeared to be more stable indicators, while the gastrointestinal symptom complex and the items *pain*, *itching* and *sweating* appeared to be less stable.

The inter-rater reliability of nurses’ ratings of symptom burden within a day showed poor and non-significant results for all items but *confusion*. Because inter-rater agreement can only be high if the rating objective remains constant, this result may be regarded as another indicator of fluctuations of symptom intensity within a day. Therefore, high-frequency documentation of symptom burden appears to be a reasonable approach. In contrast to other items, *confusion* appeared to be a stable symptom with high inter-rater agreement.

This result indicates that there was no systematic adoption of prior ratings within the instrument. If this had been the case, the interrater-agreement would have been high and significant.

### Sensitivity to change

Another matter of interest was the sensitivity of the PSBS to changes in symptom burden caused by interventions, a psychometric property that is often underreported in palliative care [39]. Given the hypothesis that interventions cause changes in symptom burden, the PSBS can be assumed to be sensitive for changes in symptom burden. In this context, it is probable that the symptom of *sweating* was not influenced by any intervention. All PSBS subscales (except *sweating*) and PSBS sum scores showed significant differences before and after palliative complex treatment intervention. Scores were significantly lower for most subscales. However, there was a significant increase in the psychosomatic and *itching* subscales. Whilst significant, it is unclear whether these findings have clinical relevance given that the psychosomatic symptom burden remained at a high level, *itching* remained at a very low level overall, and changes were measured after the decimal point [40]. From a clinical perspective, it is not surprising to observe a tentative increase in pruritus given the difficult and complex nature of its pathophysiology and treatment, including opioid-induced pruritus (OIP), and its increase in end-stage presentations of malignancy, cholestasis and uraemia [41, 42]. Psychological assessment in palliative care is inherently complex given the high level of confusion and low functioning of patients and the limited uptake of self-reported measures [43]. Further research is needed to establish the reasons for the significant increase in our psychosomatic symptom subscale.

### Lessons learned

The current study demonstrates that the implementation of a high-frequency proxy-based assessment of symptom burden in palliative patients is feasible and appears to be acceptable to nurses. According to the performed analyses, the PSBS is a feasible tool for the documentation of physical and psychological symptom burden with high sensitivity to changes in symptom burden but unsatisfactory reliability. The study further presents a framework for the post hoc validation of an already existing documentation tool to encourage other clinicians and researchers to evaluate existing documentation tools to contribute to the demand for valid and reliable outcome measures in palliative care. Based on the experiences gained during the study/the experiences authors had during the study, the authors want to share the following recommendations for further endeavours.

### Limitations

The present study deals with proxy-based measurements of symptom burden in palliative patients. Even though there are many advantages of this assessment approach, the rating itself is, to a great degree, dependent on the raters’ impression and extends only limited consideration to patients’ perception of their symptom burden. It should be mentioned that there could also have been a bias in nurses’ ratings because they were not blinded to the intervention of the palliative complex treatment. Due to the post hoc design and field setting of the study, it was not possible to use blinded raters.

Our evaluation of psychometric properties was based on classical test theory, and given our findings, it is possible that this tool is not reflective of an overall construct such as the Mini-Suffering State Examination [44] and the Palliative Outcome Scale [POS, [45]. Similar to the POS, the PSBS captures three factors and some independent items that do not load onto these factors, which makes this measure less ideal for the assessment of internal consistency and factor structure. Consequently, it appears that the PSBS is, in its present form, less suitable for this type of assessment.

In the current study, a post hoc psychometric analysis of an existing expert-developed documentation tool was performed. From a methodological perspective, a post hoc validation has its limitations. If possible, ad hoc theory-based test construction and validation should always be preferred. Further research is needed to enhance PSBS. For example, it would be an interesting research question to assess whether it can be adapted to other time scales than the prior 8 h.

### Recommendations

Regarding the complex issues of designing and/or implementing high-frequency proxy-based symptom measurement instruments, the authors recommend integrating nursing staff into the implementation process at an early stage. This integration includes offering specific training in the use of the documentation interface in addition to the possibility of providing feedback and adapting the measurement system to foster its ease-of-use. Based on the experience of the authors, this procedure increases acceptance and compliance of the measurement approach.

From a methodological perspective, the use of an expert-developed tool caused several challenges regarding the psychometric evaluation of the documentation system. Clinical experts rarely consider theoretical aspects in the development of documentation systems or measurement instruments, resulting in different measurement levels for sub-items. In the present study, it became necessary to adjust graduations of the item *pain* to ensure its comparability to other items of the PSBS. It was further necessary to exclude the item *constipation* from analyses because of its non-ordinal level of measurement. To avoid methodological challenges regarding the psychometric and clinical evaluation of patient data, the authors recommend ensuring that sub-items are measured on at least ordinal verbal rating scale with comparable intervals between characteristic values, e.g., such as the Likert scale.

To maintain the possibility of evaluating a proxy-based measurement system with respect to its psychometric properties, it is highly recommended to add an empirically validated instrument for data collection. When evaluating such instruments for their suitability, it is important to consider a similar outcome objective and comparable item structure. From a test-theoretical perspective, it is also important to assure continuous and comparable measurement times of the second instrument to maintain the possibility of evaluating construct validity at several times of measurement.

The current study yielded evidence that symptom burden is subject to frequent fluctuations in its intensity within a day. Therefore, the authors highly recommend a high-frequency measurement approach of symptom burden data. Even though this approach leads to an additional workload for nursing staff, the experience gained within this study shows that it is feasible and accepted by nurses.

## Conclusions

High-frequency proxy-based symptom burden assessment is a feasible and acceptable approach for nurse-led assessments of symptom burden in palliative care. PSBS in its present form demonstrates good structural and construct validity and high sensitivity to changes in symptom burden, but unsatisfactory reliability. This study supports the notion that PSBS might not be reflective of an overall construct and will therefore require further development and critical comparison to other already established symptom burden instruments in palliative care. Future research should focus on improving longitudinal psychosomatic symptom burden assessments.

## Abbreviations

CFA: Common Factor Analysis
ECOG: Eastern Cooperation Oncology Group
ESAS: The Edmonton Symptom Assessment System
HOPE: Hospice and Palliative Care Evaluation checklist
M: Mean
OIP: Opioid-Induced Pruritus
PCA: Principal Component Analysis
POS: Palliative Outcome Scale
PSBS: Palliative Symptom Burden Score
SD: Standard Deviation
SPCU: Specialised Palliative Care Unit
